# Influence of drug shortages on the well-being at work of pharmacists practicing in community pharmacies

**DOI:** 10.1016/j.rcsop.2024.100471

**Published:** 2024-07-02

**Authors:** Beuriot Juliette, Crunenberg Robin

**Affiliations:** a Faculty of Medicine, Department of Public Health, University of Liège, Liège, Belgium; b Faculty of Medicine, Department of Pharmacy, Center for Interdisciplinary Research on Medicines (CIRM), University of Liège, Liège, Belgium

**Keywords:** Qualitative research, Shortage, Drug, Well-being, Pharmacist, Community pharmacy

## Abstract

**Introduction:**

The problem of drug shortages is not new, but it has reached unprecedented levels in recent years. In community pharmacies, pharmacists are forced to develop daily strategies to deal with such shortages and ensure patient care. These efforts result in significant constraints and adjustments to pharmacists' daily practices. The aim of this study is to explore the possible relationship between the consequences of drug shortages and the well-being of pharmacists in pharmacies.

**Material & method:**

This study adopts an exploratory qualitative approach by interviewing pharmacists working in community pharmacies in Wallonia. The data were collected between March and June 2023 through individual semi-structured interviews using a resolute guide. The interview guide was adapted as the interviews progressed and according to the pharmacists' views. 16 participants were included, including 7 owner pharmacists, 3 non-owners, and 6 non-titular pharmacists. The interviews were transcribed and then analyzed through a thematic approach.

**Results:**

An in-depth study of the day-to-day reality of pharmacies that is open to the public highlights the time-consuming nature of drug shortages, with various implications for pharmacists' relationships, finances, and workload. However, these professionals also highlight the recognition of patients when a solution is discovered, with some sources saying that shortages value the pharmacist's ability and enhance the profession. Finally, about the possibility of change in the training of pharmacists is also addressed by some pharmacists.

**Conclusion:**

Drug shortages demand changes in pharmaceutical practice and appear to affect the well-being of pharmacists in public settings. However, the impact seems complex and is amplified by the lack of personnel. With shortages continuing to rise in recent years, it would be wise to analyze the longer-term effects of this phenomenon.

## 1 Introduction

In 2016, the World Health Organization (WHO) set up two universal definitions to explain drug shortages. These definitions distinguish between the supply of medicines and their demand. A shortage in supply occurs when the supply of essential medicines is considered insufficient to meet public health needs. On the demand side, a shortage is defined as a situation where demand exceeds supply at any point in the supply chain, potentially leading to a stock-out at the proper point of service if the cause cannot be resolved quickly.^1^

The causes of drug shortages are diverse, ranging from production problems and supply chain interruptions to regulatory constraints, quotas imposed by manufacturers, unforeseen events such as natural disasters, and economic factors. These economic factors are illustrated as follows: health insurance policies and pricing practices.^2^^,^^3^ In high-income countries, shortages are often linked to manufacturing problems,^4^^,^^5^ while in low- and middle-income countries, they may result from shortages of raw materials, drug trafficking, difficulties in obtaining manufacturing licenses, and government policies.^6^

The consequences of these shortages are wide-ranging, affecting both patients and pharmacists alike. Within Europe, a survey of twenty-nine member countries, including Belgium, was conducted by the Pharmaceutical Group of the European Union (PGEU) in 2022, which revealed that all countries have experienced drug shortages since 2019. Most countries reported a constant worsening of the situation. All classes of medicines are affected, including cardiovascular medicines, medicines for the nervous system, and anti-infectives, for instance.^7^ The consequences go beyond the material aspects, generating socio-psychological and economic effects on patients and pharmacists. The survey of the PGEU highlights the negative consequences for patients, such as emotional distress, treatment interruptions, higher costs, and suboptimal treatments. Pharmacists are also affected by financial losses, increased administrative tasks, reduced patient confidence, and reduced job satisfaction.^2^

In Belgium, drug shortages continue to increase, with repercussions for the physical and mental health of patients and pharmacists.^2^ Measures have been taken, such as a notification system to help pharmacists deal with these situations,^7^ but the number of unavailable medicines still is high,^8^ requiring continuous management by healthcare professionals, which can be time-consuming.^9^

In community pharmacies, pharmacists tend to be forced to adjust their daily activity, resulting in longer working hours, possible financial insecurity, and a blurring line between private and professional life.^2^ These factors have an impact on the well-being at work, leading to an increased mental workload, heightened obligations, and professional limitations.^1^ At a time when psychological disorders contribute sigsurveyantly to long-term sick leave,^10^ it is becoming crucial to analyze the impact of drug shortages on the well-being at work of retail pharmacists. The goal of this study is to explore how drug shortages influence the well-being of pharmacists in community pharmacies. The need for medication plays a key role in patient care. It's important to realize that pharmacists play a major role in ensuring that patients have access to their treatment, and that shortages have a major impact on the practice of their profession, which is an essential part of the patient's care pathway.^11^

## 2 Materials and methods

This study was conducted as part of the final year of a master's degree in public health at the University of Liège, following a bachelor's degree in medical laboratory technology. The participants are pharmacists practicing in different provinces of the French-speaking part of Belgium.

### 2.1 Study design

The study adopts an exploratory and inductive qualitative approach to better understand the experience of pharmacists facing drug shortages in community pharmacies, with a focus on well-being at work A qualitative approach was used to generate rich results and provide significant findings and recommendations for both training and professional activity.

### 2.2 Target population and recruitment

The target population includes pharmacists practicing in community pharmacies, both men and women, with a variety of professional experiences and employment profiles, whether as owner-operators or non-owner-operators of a community pharmacy, a group, or a chain. The geographical location of professional practice is divided into 3 categories: rural, semi-rural, or urban. The number of respondents is related to reaching the data saturation point specific to the qualitative study. Participants were recruited from across the provinces of Wallonia, using criteria such as the ability to express themselves in French and repeated experience of drug shortages.

Data collection took place over 4 months, from the end of March to the beginning of July, after approval from the ethics committee of the Centre Hospitalo-Facultaire de Liège. Initially, an online socio-demographic questionnaire was used to obtain general information about the participants, including gender, age, pharmacy location (rural, semi-rural, urban), type of pharmacy, pharmacist status, professional experience, and repeated experience of drug shortages.

Pharmacists were first contacted by e-mail, during which the researcher introduced herself and explained the research project. Some of them expressed an interest in taking part and completed the form. The pharmacist who showed interest took part in individual semi-structured interviews led by student Juliette Beuriot, which were conducted either in person or via Zoom software, based on the participants' preferences. An interview guide (annex 1), based on theoretical themes from the first literature review, was used to structure the interviews. The interview guide was also adapted as the interviews progressed and according to the pharmacists' views. This guide, which encouraged open discussion, evolved over the course of the interviews in response to participants' comments. On average, the interviews lasted between 25 and 35 min and were recorded by using the dictaphone function of a smartphone. The recordings were anonymized at the end of the interview using a code.

Below are tables describing the profiles of participating pharmacists.

The sampling method adopted was deliberately non-probabilistic, aiming for a reasoned selection of individuals to ensure a variety of experiences. The snowball sampling technique was applied following interactions with the pharmacists without pre-establishing the sample size. Following discussions with some pharmacists, they provided contact information for fellow pharmacists, and some agreed to take part in turn.

The number of participants was decided by reaching data saturation, showing that the most recent interviews provided little or no added information compared with the earlier interviews.

### 2.3 Parameters studied

**Fig. 1:**
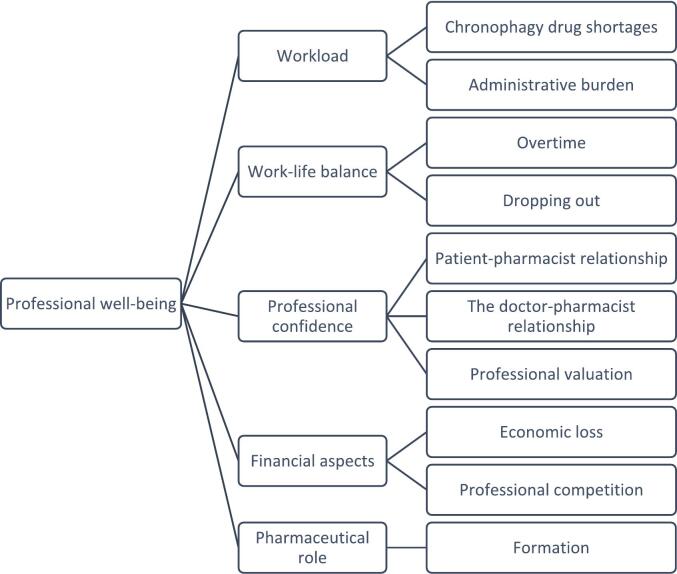
Parameters studied by the analysis model developed:

For this study, the analysis model was developed based on exploratory readings from the National Library of Medicine or Google Scholar (Fig. 1), in which points require in-depth exploration due to a lack of available information on the subject. The parameters studied were chosen based on the literature review about the influence of drug shortages in pharmacy. The identification of concepts during this review led to the formulation of a first hypothesis concerning the impact of drug shortages on pharmacists' well-being at work, with a specific focus on community pharmacies. As the data collection progressed, other themes appeared from the participants' comments. The dimensions studied were workload, work-life balance, professional confidence, financial aspects, and pharmacy training. Theses dimensions were enriched (such as the work relationship between medicine and the pharmacist) and appeared (such as the question of pharmacy training) from the interviews with pharmacists, resulting in the latest version of the interview guide (annex 1). (See Table 1, Table 2, Table 3.)

**Table 1:** Profile of the study population of pharmacists who own a community pharmacy. This person assumes primary responsibility for all operations conducted by the dispensary, including responsibility for the quality of medicines and other health products dispensed, and is the owner of the dispensary^12^ (*n* = 7).

Subject	Gender	Age (years)	Pharmacy type	Pharmacy location	Pharmacist status	Professional experience (years).
TP1	M	66	Independent pharmacy	Urban	Owner	43
TP2	M	33	Independent pharmacy	Semi-urban	Owner	10
TP3	F	54	Independent pharmacy	Urban	Owner	30
TP4	M	39	Independent pharmacy	Urban	Owner	15
TP5	F	57	Independent pharmacy	Urban	Owner	23
TP6	F	41	Independent pharmacy	Semi-urban	Owner	14
TP7	F	37	Independent pharmacy	Semi-urban	Owner	13

**Table 2:** Profile of the study population of pharmacists who do not own a community pharmacy. This person assumes primary responsibility for all operations conducted by the dispensary, including responsibility for the quality of medicines and other health products dispensed, but is not the owner of the pharmacy^12^ (*n* = 3).

Subject	Gender	Age (years)	Pharmacy type	Pharmacy location	Pharmacist status	Professional experience (years)
PTNP1	M	50	Pharmacy chain	Rural	Non-owner holder	24
PTNP2	F	47	Pharmacy chain	Urban	Non-owner holder	27
PTNP3	F	58	Pharmacy chain	Semi-urban	Non-owner holder	35

**Table 3:** Profile of the study population of non-titular pharmacist in a community pharmacy. These non-titular pharmacists conduct a range of pharmaceutical tasks under the direct supervision of the pharmacist in charge, who entrusts them with these tasks^12^ (*n* = 6).

Subject	Gender	Age (years)	Pharmacy type	Pharmacy location	Pharmacist status	Professional experience (years)
E1	F	26	Independent pharmacy	Semi-urban	Non-titular pharmacist	1
E2	F	54	Pharmacy chain	Urban	Non-titular pharmacist	28
E3	M	24	Pharmacy chain	Urban	Non-titular pharmacist	1
E4	F	27	Pharmacy chain	Rural	Non-titular pharmacist	4,5
E5	F	24	Independent pharmacy	Urban	Non-titular pharmacist	1
E6	F	23	Independent pharmacy	Rural	Non-titular pharmacist	1

### 2.4 Data processing and analysis

A total of 7 pharmacists who owned a pharmacy, 3 pharmacists who did not own a pharmacy, and 6 non-titular pharmacists were interviewed in the process. Non-titular pharmacist helps pharmacists, they do have a university degree in pharmaceutical sciences but are hired as employees.

Firstly, the individual interviews were fully transcribed using Microsoft Word. Once transcribed, the interviews were anonymized. The recordings were then listened to again. The second listening session enabled us to gain an overview of the interviews and to familiarize ourselves with what the concerned pharmacists had to say. Initial annotations were also made on the themes covered, highlighting passages of speech that were relevant and interesting for future analysis and hence could be used as verbatims.

The data processed was subject to a thematic analysis, which was conducted manually by the first author under the supervision of the promoter due to the limited size of the sample. However, to support this manual analysis and ease the stages of categorization and highlighting of themes, aid and verification via the Provalis Research software were used. Each interview was coded, and themes were found within the participants' discourse, sometimes associated according to their complementarity. These themes were linked and supported by various verbatims.

## 3 Results

### 3.1 Workload

During interviews, pharmacists stressed the time-consuming nature of managing drug shortages. The pharmacists pointed out that the time taken to deal with patients was greater when they wished to buy one or more missing medicines, both with and without a prescription. These increased delays stem from various sources, such as explaining the shortage situation to the patient, finding solutions to deliver the missing medicine: find an alternative and see if the patient agrees with this change, contact the doctor several times if the drug has been prescribed, etc. The pharmacy curriculum or the patient's self-education may save the pharmacist time in the long term, but explanations take time in the short term. Owner-operators and non-owner-operators also report an increased administrative burden associated with stock management and strategies for dealing with shortages.

Pharmacists agree that managing shortages leads to accumulated overtime, often irrecoverable, with call-backs on their days off. Some mentioned the physical impact of intense professional activity.

*“Clearly yes, overtime, that's for sure. Because the more tasks you have, the less time you have at the counter. So, you need more people to be able to serve people [...]. So, we end up working on our days off and we don't necessarily take our holidays. [...] Personally, I've got 230 hours of overtime, and I think my non-titular pharmacist has 270. We could close the pharmacy for three months between us. [...] But, on the other hand, once we manage to take our recups and take a bit of a break or go on leave, all the pressure is off. And in fact, that's when you feel the fatigue and you can't get it back. And sometimes you get sick. That's what happens when you're in a rush, it's OK, you hold on and then you let yourself go and your body, your immune system, is fooled. [...] Sometimes we're on day off and that's when the person comes back, so the pharmacy may phone us to help with the situation.”* (E4).

### 3.2 Relationship

The relationship between pharmacists and patients was discussed, highlighting that while some patients were understanding, others were aggressive. Some patients also left due to a misunderstanding, not realizing that the pharmacist was not specifically responsible for the situation. The notion of social or linguistic barriers was also discussed, as they sometimes hinder communication on the issue, making care-access more difficult for those in need.

Some patients are aware of the shortages, but many do not understand their extent. The lack of patient education on the issue is highlighted, as well as the overstocking of certain medicines by patients. Some even talk about “pharmaceutical tourism”, where patients go out of their way to ensure that they have sufficient stock.

*“It's also about educating patients. When patients bring back expired medicines, we sometimes realize that they have 3 boxes in reserve, which is useless. As we've seen with Ozempic®, some patients go on pharmaceutical tourism, visiting several pharmacies to make sure they have a box and 5 boxes in advance, whereas if they didn't proceed in this way, there might not be this hypertensive circuit either”.* (PTNP2).

Some pharmacists also mentioned the role of the media in informing patients about pharmaceutical product shortages. This information was said to be either beneficial because it highlights the problem of shortages, or harmful to pharmacists, because it can sometimes convey incorrect information.

The crucial role that doctors play in prescribed missing medicines was pointed out, with several pharmacists taking part in discussing the essential role of the doctors contributing to such a matter. In fact, when pharmacists cannot find a solution on their own, they contact the doctor, a step that was previously described as laborious. However, pharmacists no longer use this practice and feel that contacting the doctor is a waste of time. Furthermore, doctors are not always aware of the medicines that have recently been reported missing, and it is the pharmacist who must inform the provider of the situation.

In most of the interviews conducted, the doctor's understanding of the problem of shortages was highlighted. However, in some cases, doctors are less cooperative when it comes to adapting prescriptions or collaborating with the pharmacist. The case of Ozempic®, a drug prescribed for type 2 diabetes, was mentioned several times. This drug was progressively reported as missing following the diversion of its use for weight-loss programs. Pharmacists said that patients who did not have a therapeutic use for the drug should not be prescribed it any further.

*“The phenomenon has been going on for a long time now, so doctors are used to being bothered when we can't find an equivalent ourselves. We're obliged to phone the doctor to see what he's going to replace the drug with and have a chat with him. In general, we must tell him what else we could prescribe, because they're often a bit taken aback. And sometimes, there are doctors who are fuller of themselves and don't want to understand what an out-of-stock situation is. I once had a doctor who told me I just had to find the medicine myself, and there was no way he would prescribe anything else [...] But he was a specialist. In this case, general practitioners are much more understanding.”* (TP3).

Drug shortages not only impact patient care and pharmacists but also place a significant burden on other healthcare professionals. Physicians are compelled to collaborate closely with pharmacists to devise alternatives for unavailable medications. This often involves creating new prescriptions, which can be time-consuming and requires careful consideration to ensure the safety and efficacy of the substitute medication.

### 3.3 Professional confidence

On a more positive note, pharmacists report that the shortage forces them to draw on their professional ability and scientific knowledge, thereby enhancing the value of the pharmacy profession. In addition, the evolution of the profession of pharmacist and the increased importance of pharmacy in patient care were also mentioned. However, this opinion is different perspectives. For others, it is more of a question of resourcefulness than professional ability.

*“It's true that, ten years ago, the pharmacist's profession was heavily attacked, and I think that now, with this [drug shortages], with COVID, vaccination, etc., we realize that the pharmacist's profession is one of the links in the healthcare chain that has become indispensable. So, yes, that certainly enhances the value of the profession, because without our know-how, our preparations, our, yes, know-how, whether it's magistral or our know-how to find equivalents, to know the molecules etc., the profession would not be able to survive. That's the pharmacist's know-how, and it can't be substituted, or can only be substituted to a limited extent. So, it gives value to the profession”.* (TP2).

Finally, one pharmacist mentioned the fact that non-owner and owner-operator pharmacists are more affected by the loss of patient contact following the increase in support, control, and administrative tasks within the pharmacy because of shortages. Patient contact is cited as being part of the very essence of the pharmacist's profession.

### 3.4 Financial aspects

Another key point was the economic aspect of shortages. Pharmacists, both owners and non-owners, feel that the economic loss originates from time loss when managing shortages but also because of one-off purchases of certain drugs. In addition, pharmacists choose to buy from a single wholesaler because this offers financial advantages but reduces access to a larger stock. The difficulties associated with drug shortages have also given rise to growing solidarity among pharmacists, aimed at keeping a quality service for patients. This solidarity varies according to pharmacists' social status. In some cases, a rivalry dynamic is also appearing, with continuous increases in stocks and sometimes rivalries between independents and group pharmacies.

*“It's more the time we have to take, that we spend managing all this. It's not a pharmacist's hourly rate. I mean, I don't get paid at the pharmacy for that. At the pharmacy, you get paid when you sell something. If, in the end, I don't have a solution, [...] I won't have earned anything. I might have spent all morning or all day looking for a solution that I didn't find. [...] Economically too, I only have one wholesaler because I get the best conditions by collaborating with a single wholesaler, [...] if I work with 2 wholesalers, I'll get worse conditions because that means my sales will be divided by 2 [...]”.* (TP6).

*“It's more a question of solidarity. For example, I have a pharmacist neighboring me 200 meters away. We help each other out daily. We have a common wholesaler, but we each have a wholesaler that the other doesn't have. [...] We each have a different wholesaler because that allows us to help each other out, sometimes once or several times a day. I find a product for him that is still with my wholesaler, but that he no longer has with his wholesaler, and vice versa. It is more difficult with the chains, but independents help each other out”.* (TP1).

### 3.5 Pharmacy training

Finally, some pharmacists feel that current pharmacy training no longer effectively meets the challenges facing the profession, suggesting the need for reform to better prepare students for the realities of the field. Some advocate for an earlier emphasis on pharmacy in the curriculum, while others support that training is regularly updated by universities to keep pace with developments in the profession. In other words, they feel that the overall training is adequate, stressing the importance of practical learning, which plays a crucial role as a complement to solid theoretical training.

*“The most important thing for me is to have a scientific background with a more in-depth pharmacy focus. In other words, in our training, we have an overall scientific training. Then, in the last year, we'll have some dispensary training. We need to go much further in all aspects of treatment analysis and pharmaceutical care. Here, we learn a lot during our internships, and we also learn a lot in the field thanks to the scientific baggage we accumulated during our studies. But we missed a lot of pharmacy-oriented courses in our curriculum. [...] We'd choose our orientation so we could already focus our courses [...] and then we'd have even more effective pharmacists”.* (TP2).

## 4 Discussion

### 4.1 Workload

As showed in a study of hospital pharmacies in Belgium conducted in 2017,^13^ this work confirms that drug shortages do in fact have an impact on the workload of dispensing pharmacists. Pharmacists who invest time out of their day to resolve the shortage problem are more likely to increase their responsibilities in addition to their workload. Managing such situations involves explaining the situation to patients, looking for alternatives, cooperating with doctors, manufacturers, wholesalers, and laboratories, and managing drug stocks. Although pharmacists find it difficult to provide a precise figure in terms of the hours devoted to these tasks, the estimates collected show an average of 2 to 3 h per week, less than the average of 6 h and 40 min per week reported by the PGEU.^2^

In addition, drug shortages appear to contribute to an imbalance between pharmacists' professional and private lives. Pharmacists report an accumulation of overtime due to shortages, compounded by staff shortages in the pharmaceutical sector. The shortage of pharmacists is attributed to changes in the profession, the diversification of tasks in community pharmacies, and technological advances such as electronic prescriptions. These constraints limit pharmacists' ability to take some time off or recover their overtime, and even when a break is possible, such time off does not guarantee effective rest or sufficient time off work. Yet the possibility of flexible working hours and a break from professional activity have proved to be pillars of well-being at work for both employees and self-employed workers.^14^^,^^15^

### 4.2 Relationship

Pharmacists commented on the impact of drug shortages from a psycho-social point of view, highlighting both the positive and negative effects on patient relations. Some patients, well informed about the situation, are understanding, reinforcing their trust in their regular pharmacist. On the other hand, others, unaware of the problems of shortages, may become aggressive on learning that the medicines they need are unavailable, leading some to turn to competitors. Overall, pharmacists regret the lack of patient awareness of shortages, stressing the negative impact on confidence in pharmacy professionals, an observation also corroborated by the survey conducted by the PGEU.^2^

Also, some pharmacists note the importance of the media in informing patients about drug shortages. However, they point out that media coverage of this crucial problem is not always proportionate to its importance.^9^ The media, as powerful vectors of influence,^16^ should give correct information to avoid any misunderstanding among patients and help keep confidence in pharmacists.

As far as the work relationship between pharmacists and doctors is concerned, most pharmacists describe a positive work relationship, marked by a mutual understanding of the shortage phenomenon. The responsibility of doctors in the occurrence of certain shortages was also raised, particularly in the case of Ozempic®, a drug for type 2 diabetes.

### 4.3 Professional confidence

Finally, the evolution of the profession of dispensing pharmacist has been discussed, with the profession undergoing a major transformation over the years, taking on greater responsibility and playing an.

increasingly significant role in patient care.^17^ Some pharmacists believe that these shortages reinforce the importance of pharmacists by making their ability even more indispensable. Others see solving these problems as a headache rather than an opportunity to enhance their profession.

### 4.4 Financial aspects

Pharmacists unanimously agree that most of their financial losses result from the time invested to address drug shortages, as also highlighted in the EMP report based on returns from 29 different countries.^15^ This problem has a particular impact on pharmacist owners. The hours dedicated to managing shortages are a labor cost, whether it is for an individual owner, a group, or a chain. The 2022 EMP report highlights that time spent managing shortages peaked last year,^15^ directly affecting sales. A prolonged sale following the management of a shortage results in a decrease in the total number of sales and financial instability for the pharmacy. In addition, other responsibilities within the pharmacy may be delayed or performed outside regular working hours.

On the other hand, the economic loss can also result from the unresolved shortages, prompting the patient to turn to another community pharmacy. Shortages thus reinforce the rivalry between.

pharmacists and increase the bargaining power of patients.^17^ This competition seems more marked between pharmaceutical chains and independent pharmacies. Some respondents working in pharmacy chains noted that merchandise exchanges are numerous but regulated, often limited within their own group. However, even within a structure that encourages solidarity, individualistic trends are developing in stock sharing. Some pharmacists also raise the issue of overstocking, contributing to the inaccessibility of certain drugs. On the other hand, more solidarity is appearing between independent pharmacists, who have developed collaborative strategies to ensure quality management for patients. None of the interviewees mentioned a lack of social interaction; instead, they pointed to partial collaborations aimed at solving the problem of shortages and which could serve as social support.^18^

Finally, the last source of economic loss for pharmacists is the purchase of substitutes in small quantities from certain wholesalers. This results in higher costs for the pharmacist, who loses the commercial advantages of buying in bulk, such as discounts to increase the profit.

### 4.5 Pharmacy training

During this study, some professionals raised some questions about the possibility of change in the training of pharmacists. Some suggest that pharmacy training should focus more on the pharmacy aspect in the early years of the curriculum. Others propose the creation of a sixth year, although this approach is complex to implement at the Belgian national level. It should be noted that the University of Liège is readjusting its pharmacy program, considering introducing the official aspect of the profession earlier in the curriculum.

It would also be important to encourage continuing education to help pharmacists better adapt to the evolution of the profession. Adequate preparation for unpredictability and work situations reduces stress and increases engagement and job satisfaction. Thus, encouraging the continuing training of pharmacists appears to be an essential asset to promoting their professional development.

Promoting pharmacy studies and the pharmacist profession are also important areas to address. Engaging in awareness days in secondary schools, and potentially even in primary schools, could help inspire future vocations and generate interest in the field from a young age.

## 5 Conclusion

The purpose of this study was to explore the possible correlation between drug shortages and the workplace well-being of public pharmacists. Analysis of pharmacists' discussions confirms that shortages affect their professional well-being. In other words, they lead to a particular increase in workload, unstable schedules, and confusion between private and professional life. A decrease in patient confidence and the presence of economic insecurity are also the results of this problem, marked more significantly among pharmacist owners or non-owners of a pharmacy for a chain.

Paradoxically, for some pharmacists, these shortages increase job satisfaction by valuing the profession as an indispensable link in the chain of care. However, the phenomenon of shortages seems to have intensified over the past year, justifying an exploration of the longer-term effects. It is also important to note that the impact of the shortage problem is multifactorial. The impact of drug shortages on the pharmacist is amplified by the lack of personnel. The pharmacist must manage many tasks individually, when two pharmacists would be needed to complete the tasks. Pharmacists who must manage shortages are unable to perform their day-to-day duties properly due to lack of time and would require added help which is not available on the labor market.

To help pharmacists manage shortages, certain actions are needed to improve interactions with patients or physicians as well as reduce wasted time, which should be considered. It is advisable to improve patient education on shortages through large-scale awareness campaigns, emphasizing the delicate position that pharmacists have. Physicians have a critical role to play in drug delivery and that better collaboration between the pharmacy and the practice could be beneficial.

Finally, the pharmacist profession seems to have undergone a significant evolution in recent years, requiring rapid adaptation for professionals. Adjusting training such as to better prepare them for life in a pharmacy setting and encouraging ongoing training for pharmacists in pharmacies appear to be solutions to prepare them for future challenges.

The repercussions of drug shortages on the work of pharmacists are a source of stress and financial insecurity for pharmacists daily. All types of pharmacists are affected, but in the light of this work the consequences of shortages have a greater impact on community pharmacy owners. In addition, this problem can frustrate pharmacists who are unable to practice their profession properly and constitutes a challenge and an added mental burden in the context of their profession.

## Funding

This research receives no specific grant from funding agencies in the public, commercial, or not-for-profit sectors.

## CRediT authorship contribution statement

**Beuriot Juliette:** Writing – original draft, Investigation, Formal analysis, Data curation, Conceptualization. **Crunenberg Robin:** Writing – review & editing, Supervision, Funding acquisition, Conceptualization.

## Declaration of competing interest

X The authors declare that they have no known competing financial interests or personal relationships that could have appeared to influence the work reported in this paper.

## Appendix A Interview guide (last version) for a qualitative study with the research question “How do drug shortages influence pharmacists' well-being at work in a pharmacy open to the public?” by Juliette Beuriot

### A.1 Introduction

Brief introductions (presentation of the researcher, reminder of the research project, etc.).

Renewal of application for registration authorization.

Reminder of speech rules and ethics (consent sent beforehand via email or signed on the day before the interview starts).

### A.2 Introductory statement by the interviewer on the topic of work:

#### A.2.1 Opening questions

Can you narrate an impactful experience/a situation that reminds you of medication shortages?

#### A.2.2 Questions

Have you noticed a change in the pace of your profession in recent years? Why? What do you think about the increase in pharmacist services?

In your opinion, what are the direct and indirect consequences of these shortages in managing your pharmacy? (economic, relational, temporal, professional trust, etc.).

Could you explain the nature of the relationships you have with your patients about medication shortages (trust, good relationship, mediocre, etc.)?

Can you describe how the relationship with doctors unfolds in the context of medication shortages (understanding, conflict, communication, etc.)?

Have you implemented personal strategies to combat shortages? What are they? (Themes: competition/solidarity among pharmacists + themes: time-consuming nature of shortages).

Have you relied more on your scientific skills and knowledge in recent years due to the increase in shortages (confidence/professional satisfaction)?

Do you think the current training of pharmacists is still suitable for the challenges related to the profession? If not, how would you adapt it?

### A.3 Closing

Summary/brief conclusion of the information gathered during the interview.

Feedback on the interview experience.

Final open question: As we come to the end of this interview, is there anything important you would like to address that we may have missed, or something you would like to revisit?

Thanks.

## References

[1] Abaidi J., Drillon D. (2017). Les dimensions du bien-être au travail: Axes de prévention des risques psychosociaux?. Rev Intern Psychosociol Gest Des Comport Organisat.

[2] Pharmaceutical Group of the European Union (2022).

[3] Shukar S., Zahoor F., Hayat K. (2021). Drug shortage: causes, impact, and mitigation strategies. Front Pharmacol.

[4] Dill S., Ahn J. (2014). Drug shortages in developed countries—reasons, therapeutic consequences, and handling. Eur J Clin Pharmacol.

[5] Ventola C.L. (2011). The drug shortage crisis in the United States: causes, impact, and management strategies. P & T: A Peer-Rev J Formul Manage.

[6] Khan F.A. (2020). Anesthetic drugs shortage in lower- and middle-income countries: a safety and quality issue. Anaesth Pain Intens Care.

[7] Claus B., Pauwels K., Baert M. (2015). Drug shortages in the hospital: management, causes and budget impact. J Pharm Belg.

[8] Agence fédérale des médicaments et produits de santé. (n.d.). PharmaStatut, L'application en ligne pour vérifier la disponibilité des médicaments [Computer software]. Retrieved 25 January 2024, from https://pharmastatut.be/.

[9] Bogaert P., Bochenek T., Prokop A., Pilc A. (2015). A qualitative approach to a better understanding of the problems underlying drug shortages, as viewed from Belgian, French and the European Union’s perspectives. PLoS One.

[10] Zanardelli M., Genevois A.-S., Mazoyer T. (2011).

[11] Tsuyuki R., Bond C. (2019). The evolution of pharmacy practice research-part I: time to implement the evidence. Int J Pharm Pract.

[12] Association Pharmaceutique Belge. (n.d.). L'équipe de la pharmacie. Consulted 23 March 2024, from https://www.apb.be/fr/corp/sante-publique/La-pharmacie/L-equipe-de-la/pharmacie/Pages/default.aspx.

[13] De Weerdt E., De Rijdt T., Simoens S., Casteels M., Huys I. (2017). Time spent by Belgian hospital pharmacists on supply disruptions and drug shortages: an exploratory study. PLoS One.

[14] Bellemare M. (2010). Organisation pathogène du travail et maintien durable en emploi: Une question antinomique? Sous la direction de Marie-France Maranda et Geneviève Fournier, Québec: Presses de l’Université Laval, collection Trajectoires professionnelles et marché du travail contemporain, 2009, 228 p. Relat Indust.

[15] Direction de l'’animation de la recherche, des études et des statistiques (DARES) (2009).

[16] Ben Youssef I. (2018). Grégory DERVILLE (2017), Le pouvoir des médias: 4e édition, Grenoble, Presses universitaires de Grenoble, Coll. « Politique en plus ». Communication.

[17] Reyes G. (2023). La complexité du métier de pharmacien titulaire d’officine. @GRH, N°.

[18] Celerier S. (2013).

